# miRNA regulation in the early development of barley seed

**DOI:** 10.1186/1471-2229-12-120

**Published:** 2012-07-28

**Authors:** Julien Curaba, Andrew Spriggs, Jen Taylor, Zhongyi Li, Chris Helliwell

**Affiliations:** 1 CSIRO Plant Industry, GPO Box 1600, Canberra, ACT, 2601, Australia

**Keywords:** microRNA, Barley, Grain development, Plant hormones, Disease resistance, Small RNA sequencing, mRNA degradome, PARE

## Abstract

**Background:**

During the early stages of seed development many genes are under dynamic regulation to ensure the proper differentiation and establishment of the tissue that will constitute the mature grain. To investigate how miRNA regulation contributes to this process in barley, a combination of small RNA and mRNA degradome analyses were used to identify miRNAs and their targets.

**Results:**

Our analysis identified 84 known miRNAs and 7 new miRNAs together with 96 putative miRNA target genes regulated through a slicing mechanism in grain tissues during the first 15 days post anthesis. We also identified many potential miRNAs including several belonging to known miRNA families. Our data gave us evidence for an increase in miRNA-mediated regulation during the transition between pre-storage and storage phases. Potential miRNA targets were found in various signalling pathways including components of four phytohormone pathways (ABA, GA, auxin, ethylene) and the defence response to powdery mildew infection. Among the putative miRNA targets we identified were two essential genes controlling the GA response, a *GA3oxidase1* and a homolog of the receptor *GID1*, and a homolog of the ACC oxidase which catalyses the last step of ethylene biosynthesis. We found that two *MLA* genes are potentially miRNA regulated, establishing a direct link between miRNAs and the R gene response.

**Conclusion:**

Our dataset provides a useful source of information on miRNA regulation during the early development of cereal grains and our analysis suggests that miRNAs contribute to the control of development of the cereal grain, notably through the regulation of phytohormone response pathways.

## Background

MicroRNAs (miRNAs) are a class of non-coding small RNAs (smRNAs) that act to reduce expression of target genes by interacting with their target mRNAs in a sequence-specific manner. Since their discovery it has become clear that miRNAs are an important component in the regulation of many genes in most eukaryotic cells. In plants, most currently validated miRNA targets code for transcription factor families with crucial developmental functions, including the control of root and shoot architecture, vegetative to reproductive phase transitions and leaf and flower morphogenesis [1,2].

miRNAs are processed from a primary miRNA transcript which folds to form an imperfect stem-loop. The pri-miRNA hairpin is recognised and processed to a smRNA duplex consisting of the miRNA and complementary miRNA* by a protein complex containing a DCL1-type RNase. The mature miRNA, which is typically 20–21 nt in length, is then incorporated into the RNA Induced Silencing Complex (RISC) to regulate one or more target genes *in trans* through a base pairing mechanism. Most plant miRNAs appear to trigger both mRNA cleavage (between the nucleotides matching the 10^th^ and 11^th^ position of the miRNA) and translational repression of their target genes [3]. Although these two mechanisms are additive, they can be dissociated when slicing activity is disabled by a mis-pairing in the central region between the miRNA and its target [4-7]. In plants, the high level of complementarity between the miRNAs and their targets suggests slicing is the predominant mode of action of miRNAs [7]. Alternatively, miRNAs can regulate their target indirectly through the production of trans-acting short interfering RNAs (tasiRNAs) [8,9]. tasiRNAs are synthesised from a non-coding mRNA that is processed to phased 21 nt smRNAs by a miRNA triggered process. Like miRNAs, tasiRNAs can regulate multiple target genes through a slicing mechanism.

The number of annotated miRNAs in miRBase has exponentially increased in the last decade [10]*.* The earliest group of miRNAs were identified *in silico* using algorithms to predict stem-loop precursors and targets present in the genome and/or EST databases [11-15]. Subsequent developments in high throughput sequencing made it possible to identify miRNAs based on sequencing of smRNA libraries in a wide range of species. Schreiber *et al.*[16] identified 100 miRNAs, including 44 new miRNAs, from barley leaves using short-read sequence data. A major challenge of sequencing based approaches is to identify the miRNAs amongst a smRNA population mostly composed of short-interfering RNAs (siRNAs) [17]. Distinguishing these two major smRNA classes relies principally on identifying their origin. An siRNA locus produces several overlapping siRNAs, whereas the pri-miRNA encoded by a *MIR* gene usually produces one miRNA from an imperfect RNA hairpin [18]. Additional criteria can also help classify a smRNA, such as its length and mode of action. Most miRNAs and tasiRNAs are 21 nt in length and post-transcriptionally regulate their target genes *in trans*, whereas the vast majority of the 24 nt smRNAs correspond to cis-acting siRNAs (casiRNAs) that regulate the transcription of their own locus of origin through a DNA methylation based mechanism.

miRNA targets are often validated using a modified 5’RACE technique to detect the products of miRNA-mediated cleavage [19]. For most currently annotated miRNA targets, cleavage has not been verified and therefore the function of the corresponding miRNA *in vivo* has not been established. Recently, techniques which combine 5’RACE and high throughput sequencing (Parallel Analysis of RNA Ends (PARE) and equivalent methods [20-22]), have been used to simultaneously validate all sliced miRNA targets in a given RNA extract. Such an approach has been successfully carried out in Arabidopsis, rice, soybean, grapevine, citrus and medicago [23-28]. However, identifying a miRNA regulation is dependent on examining the appropriate tissue and developmental stage. As miRNAs are predominantly post-transcriptional regulators [29,30], the impact of their regulation depends on the overlap of their spatio-temporal expression with that of their target genes [1,2]. miRNAs from the same family can potentially have different functions depending on their expression profile, as suggested for members of the miR169 and miR171 families that differentially accumulate in response to abiotic stress in rice [31,32].

Despite the growing knowledge of miRNA functions in plants, only the functions of highly conserved miRNAs have been investigated in crop species. Perhaps the best characterized miRNAs in cereals are miR156 and miR172 which regulate *SPL* (*Squamosa Promoter-binding protein-Like*) and *AP2*-like genes, respectively. miR156 controls shoot branching in rice and maize [33-35] and miR172 regulates floral organ identity in rice, maize and barley [36-41]. In maize, miR172 accumulation is affected by miR156 and both miRNAs are involved in the regulation of the juvenile to adult phase transition [33]. In contrast to the highly conserved miRNAs, the majority of the newly discovered miRNAs are weakly expressed and only found in closely related species, suggesting that they have recently evolved and could contribute to determining species-specific traits.

Barley is the fourth most cultivated crop worldwide; its grains are used for both human consumption and livestock feed. From anthesis, it takes approximately 40 days to form a mature grain composed of 3 principal tissues: the embryo, the endosperm (starchy endosperm and aleurone layers), and the outside layer (seed coat and pericarp). The development of the grain can be divided in three principal stages based on morphological changes, metabolite accumulation and transcriptome analysis: pre-storage, storage (or maturation) and desiccation [42-45]. The pre-storage phase, which corresponds to the first 5 Days Post Anthesis (DPA), is characterized by extensive mitotic activity in both embryo and endosperm. The transition to the storage phase, roughly between 5 and 10 DPA, can be considered as an intermediate stage characterized by dramatic transcriptional changes in order to mobilize energy resources and initiate the differentiation of the tissues that will constitute the mature grain. Throughout the maturation phase, which lasts up to ~25 DPA, aleurone and embryonic tissues acquire desiccation tolerance whereas the endosperm cells undergo endoreduplication and accumulate storage metabolites (mainly starch and proteins) [46].

In this study we investigated the miRNA-mediated gene regulation that takes place during the growth of the barley grain. Since the early stages of development play a key role in determining grain quality characteristics, we focused on the pre-storage and early storage phases (0–15 DPA). From analysis of smRNA and degradome libraries, 96 genes regulated by miRNA-mediated cleavage were identified including transcription factors, kinases, oxidoreductases, hydrolases, transferases, receptors and transporters. Our data suggest that miRNAs contribute widely to the control of development of the cereal grain, notably through the regulation of phytohormone response pathways.

## Results and discussion

**Figure 1:**
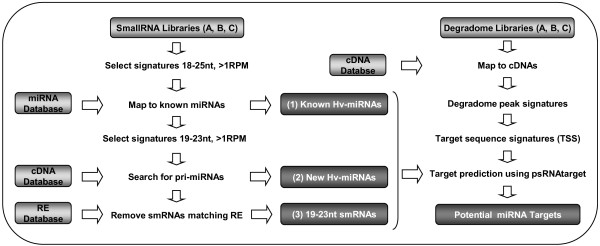
**Flow diagram of smallRNA and degradome library analysis.** Three smRNA libraries and three mRNA degradome libraries were made from whole barley caryopses at three developmental stages: A (1–5 DPA), B (6–10 DPA) and C (11–15 DPA). Light grey boxes indicate the input data (RE stands for Repeat Elements) and dark grey boxes represent the output data. The (1) known miRNAs, (2) new miRNAs and (3) 19-23nt smRNAs identified from the analysis of the smRNA libraries were then used in combination with the degradome libraries to find potential miRNA regulated genes.

The early development of the seed is marked by large-scale transcriptional changes, especially during the transitional phase. In order to correlate those changes with variation in miRNA abundance, we made smRNA and mRNA-degradome libraries from the whole caryopsis at three consecutive developmental stages: (A) from 1 to 5 DPA (early pre-storage), (B) from 6 to 10 DPA (late pre-storage or transition phase), and (C) from 11 to 15 DPA (early storage). An overview of our analysis is presented Figure 1. We first used the smRNA libraries to detect known miRNAs and to identify new miRNAs based on the presence of their precursor in cDNA databases. We then used the degradome libraries to identify potential endonuclease cleavage sites in EST sequences and selected those that could result from slicing by a sequenced smRNA. The smRNAs associated with a cleavage site in the degradome data are designated as potential miRNAs (pot-miRNAs).

### Diversity of the small-RNA population in early grain development

**Figure 2:**
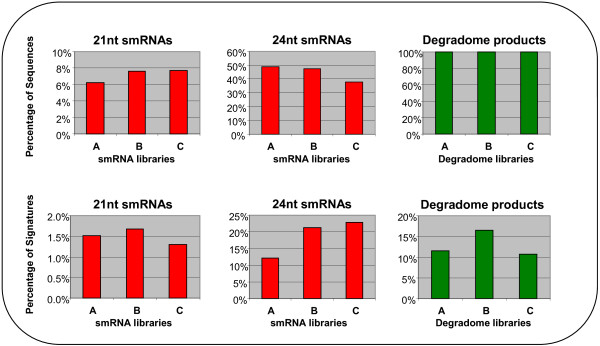
**Distribution of the 21 nt-smRNAs, 24 nt-smRNAs and degradome products by library.** The percentages of sequences and signatures were calculated using the total number of sequences for each library (A, B, C). Degradome products correspond to the 20-21nt sequences present in the mRNA degradome libraries.

Approximately equal numbers of sequence reads (20 million) were generated from each of the smRNA libraries (Table 1, Additional file 1). The size distribution in the smRNA datasets was similar to previous reports with about 44 % 24 nt sequences that are likely to consist predominantly of casiRNAs and 7 % 21 nt smRNAs that will include the bulk of the miRNAs (Figure 2). The datasets showed a decrease in the percentage of 24 nt smRNAs and an increase in the percentage of 21 nt smRNAs from stages A to C, which correlates with data from developing rice grain samples from 1–5 DPA and 6–10 DPA [47]. If unique signatures are considered, both 21nt and 24 nt smRNA diversity increased from stage A to B, suggesting a higher smRNA complexity during the reprogramming phase of grain development. As the grain matures further (sample C), the number of unique 21 nt signatures decreases while the 24 nt increase (Figure 2). The continuing increase in 24 nt smRNA diversity with development may reflect an increase in heterochromatin formation as cells become more differentiated. This correlates with the observation that undifferentiated cells have little heterochromatin and that epigenetic regulation plays an important role in the determination of cell fate through global remodelling and compaction of chromatin structure [48,49].

**Table 1:** Composition of the smRNA libraries

**smRNA length**	**Sequences (million)**				**Signatures (million)**						
	**A**	**B**	**C**	**Total**	**A**	**B**	**C**	**Total**	**Singletons**		**>1RPM**
1-36 nt	6.608	6.567	6.904	20.079	1.511	2.251	2.397	6.159			
18-25 nt	5.284	5.214	4.837	15.335	1.336	2.046	2.159	4.801	3.900	81%	0.137
19-23 nt	1.682	1.806	1.910	5.398	0.449	0.560	0.507	1.313	1.080	82%	0.041
21 nt	0.410	0.500	0.530	1.440	0.100	0.110	0.090	0.249	0.200	80%	0.008
24 nt	3.240	3.100	2.590	8.930	0.800	1.400	1.570	3.268	2.640	81%	0.090

Number of sequences and signatures (unique sequences) for different length of smRNAs found in smRNA libraries made from whole caryopsis tissue at the three developmental stages: A (1–5 DPA), B (6–10 DPA) and C (11–15 DPA). Singletons are the signatures found only once. The column “>1 RPM” shows the number of signatures with more than 1 Read Per Million (RPM).

### Previously identified miRNAs present in barley grain

**Figure 3:**
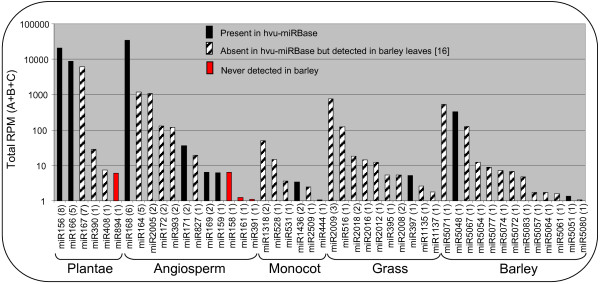
**Known miRNA families found in the barley seed.** miRNA families were grouped according to their conservation level across the plant kingdoms (based on the farthest species from barley in which at least one member has been found). For each miRNA family, the number of members (or unique sequences) identified in barley is presented in brackets and the total number sequences from the three libraries is presented in reads per million (RPM). In black are the families for which at least one member has been classified in miRBase, in black stripes are the miRNA families not classified in miRBase but previously detected in barley leaves [16] and in red are the known miRNA families absent in barley leaves.

We found 84 smRNA signatures that were identical (in sequence and length) to at least one previously identified plant miRNA, representing 47 miRNA families (Figure 3, Additional file 2). Of these, 11 families had been previously classified as hvu-miRNA in miRBase and 32 were previously reported in barley leaves but not classified as hvu-miRNA in miRBase [10,16]. We found 4 miRNA families (hvu-miR894, hvu-miR158, hvu-miR161, hvu-miR391) that were not observed in barley leaf and so may be seed specific [16] (Figure 3, Additional file 2). As previously observed by Colaiacovo et al. [50], the vast majority of the miRNAs are 21 nt in length (Additional file 2). The specificity of each family was determined according to the farthest species (from barley) in which at least one member has been found. We note that the highly conserved families are not necessarily highly expressed in barley seed, examples are miR894 and miR408 which accumulate at less than 10 RPM. Conversely, miR5071, miR5048 and miR5067 which have only been identified in barley, are expressed at over 100 RPM in the seed. Overall only ~0.01% of the unique 21 nt signatures correspond to known miRNAs.

**Figure 4:**
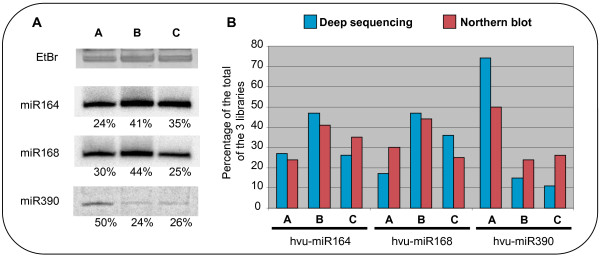
**Level of mature miRNAs in the smRNA libraries*vs in planta.*A**. Northern blot showing the level of mature miRNAs in whole caryopsis tissues harvested from three stages: 1–5 DPA (A), 6–10 DPA (B) and 11–15 DPA (C). The same amount of total RNA was loaded based on spectrophotometer readings. The intensity of each band was quantified using a phosphoimager and is indicated as a percentage. **B**. Distribution of three known miRNAs between the three stages (A, B, C) using read numbers in the three smRNA libraries (blue) and their abundance detected by northern blot in 5A (red). The percentages for each miRNA refer to the total abundance or reads of the miRNA detected across the three samples or libraries.

To determine whether the number of cloned sequences in the libraries reflects the relative abundance of a smRNA *in planta*, the accumulation of three known miRNA families (hvu-miR164, hvu-miR168 and hvu-miR390) was monitored during seed development (Figure 4). For all three families, the abundance of the mature miRNA detected by northern blot between the three development stages followed the same trend as the numbers of reads in the libraries. Therefore, the relative expression of each smRNA between the 3 samples (A, B and C) can be directly inferred from the numbers of sequence reads.

### New miRNAs identified based on the presence of their precursor

A miRNA can potentially evolve as a result of the transcription of one of the many inverted repeats present in the genome if the resulting hairpin structure has the features to be recognised and processed by a DCL protein. In the absence of a barley genome sequence, sequence information is restricted to EST databases. For this analysis we used the HarvEST database which contains over 50,000 unigenes and searched for miRNA precursors corresponding to smRNA sequences in our database (Figure 1). A putative precursor (pri-miRNA) was found for 15 smRNA sequences. Eight of these were for miRNAs present in miRBase including the three highly conserved miRNAs hvu-miR159, hvu-miR171a and hvu-miR168a for which a miRNA* was also present (Table 2, rows 1–8; Additional file 3). There were also putative pri-miRNAs for seven smRNA sequences not present in miRBase (Table 2, rows 9–15). Two of these have sequences closely related to known miRNA families and were therefore annotated hvu-miR5071b and hvu-miR1120b. Hvu-miR1120b (21 nt) is a short version of hvu-miR1120 (24 nt) with 3 nucleotides missing at the 5’ end; both are predicted to originate from the same pri-miRNA. Since hvu-miR1120 was only predicted *in silico*[51] and hasn’t been detected in barley leaves, it may not exist *in planta*. We temporarily annotated the other five smRNAs as new miRNAs starting from hvu-miR6001 as they show the expected features of a miRNA other than the presence of a miRNA*. The lack of miRNA* sequences may reflect the low abundance of these miRNAs.

**Table 2:** miRNA precursors and corresponding known and new miRNAs

**Row**	**EST ID**	**pri-miRNA**	**miRNA**	**miRNA sequence (5’-3’)**	**Length**	**RPM-A**	**RPM-B**	**RPM-C**	**Star**
1	U21_20916	hvu-MIR159a	hvu-miR159	TTTGGATTGAAGGGAGCTCTG	21	2.74	2.16	1.29	YES
2	U21_9346	hvu-MIR168	hvu-miR168a	TCGCTTGGTGCAGATCGGGAC	21	5891.89	16016.44	12430.78	YES
3	U21_38648	hvu-MIR171	hvu-miR171a	TGATTGAGCCGTGCCAATATC	21	13.41	5.13	3.87	YES
4	U21_6953	hvu-MIR444a	hvu-miR444a	TTGTGGCTTTCTTGCAAGTCG	21	0.41	0.41	0.26	NO
5	U21_50801	hvu-MIR5051	hvu-miR5051	TTTGGCACCTTGAAACTGGGA	21	0.41	0.54	0.39	NO
6	U21_37849	hvu-MIR1135	Hvu-miR1135	TGCGACAAGTAATTCCGGACGGAG	24	0.27	0.68	1.68	NO
7	U21_8425	hvu-MIR1436b	hvu-miR1436b	TACATTATGGGACGGAGGGAG	21	0.82	1.08	0.26	NO
8	U21_2275	hvu-MIR5048	hvu-miR5048	TATTTGCAGGTTTTAGGTCTAA	22	111.82	122.18	89.92	YES
9	U21_14449	hvu-MIR5071b	hvu-miR5071b	TCAAGCATCATGTCATGGACC	21	0.55	0.54	0.26	NO
10	U21_51424	hvu-MIR6001	hvu-miR6001	CGAGGATGAAGAAGAAAAT	19	3.15	1.62	0.90	NO
11	U21_16909	hvu-MIR6002	hvu-miR6002	TAGGACGCCATGGTAGATAGCATG	24	0.00	1.22	1.03	NO
12	U21_27580	hvu-MIR6003	hvu-miR6003	AATATGGATCGGAGGGAGTAA	21	0.68	0.95	0.52	NO
13	U21_53039	hvu-MIR6004	hvu-miR6004	TTGCGTCGTTGTGCCTGGGCT	21	0.96	0.41	0.00	NO
14	U21_51897	hvu-MIR6005	hvu-miR6005	AATTAATTTGGATCGGAGGGA	21	0.41	0.27	0.65	NO
15	U21_16332	hvu-MIR1120	hvu-miR1120b	TTCTTATATTATGGGACGGAG	21	4.11	4.46	5.29	NO

The pri-miRNAs were identified in the harvEST dataset according to their ability to form a hairpin-like secondary structure that could generate one of the smRNAs present in the libraries. For each miRNA, its length and number of reads (in RPM) in each library (A, B, C) are indicated. The column “Star” indicates when a corresponding miRNA* was found in the libraries.

### Identification of potential miRNA targets using the degradome libraries

The 84 known and 7 new miRNAs identified in this study account for only 1 % of the unique 21 nt signatures in the smRNA sequence dataset, suggesting that these analyses did not identify all the miRNAs present. An alternative approach to identify the presence of a miRNA is to detect its post-transcriptional regulatory activity on a target gene. In plants, most miRNAs characterised to date show slicing activity on their target, hence degradome analysis was carried out using the Parallel Analysis of RNA Ends (PARE) technique [21], constructing libraries from samples A, B and C as used for the smRNA libraries (Figure 1). We reasoned that having smRNA and degradome libraries from the same sets of samples would allow us to follow the miRNA regulation of target genes and increase the likelihood of detecting a cleavage that occurs at a particular developmental stage.

Approximately 30 million sequence tags corresponding to cleaved 5’ ends of mRNAs were obtained from each of the degradome libraries (Table 3, Additional file 1). After trimming of adapter sequences, most sequences were of the expected size of 20 or 21 nt. To simplify analysis, the 21 nt sequences had the 3’ nucleotide trimmed and were then pooled with the 20 nt sequences giving 8.86 million unique sequence tags (Table 3). The number of unique sequences was higher in sample B which also had the greatest diversity of 21 nt smRNAs (Figure 2). This suggests that there is a larger diversity of transcripts regulated by miRNA cleavage between 6 and 10 DPA.

**Table 3:** Composition of the mRNA degradome libraries

**Degradome length**	**Sequences (million)**				**Signatures (million)**			
	**A**	**B**	**C**	**Total**	**A**	**B**	**C**	**Total**
20-36 nt	29.741	29.282	27.365	86.389	3.800	5.526	3.168	10.147
20 nt	13.150	11.949	12.103	37.203	1.724	2.393	1.411	4.502
21 nt	16.525	17.239	15.188	48.952	2.066	3.110	1.746	5.607
20-21 nt pooled	29.675	29.188	27.291	86.155	3.433	4.814	2.928	8.857

Number of sequences and signatures (unique sequences) of mRNA degradation products found in the degradome libraries made from whole caryopsis tissue at the three developmental stages A (1–5 DPA), B (6–10 DPA) and C (11–15 DPA). The 20–21 nt pooled degradome sequences combine the 20 nt and 21 nt sequences shortened to 20 nt by removing the 3’end nucleotide.

**Figure 5:**
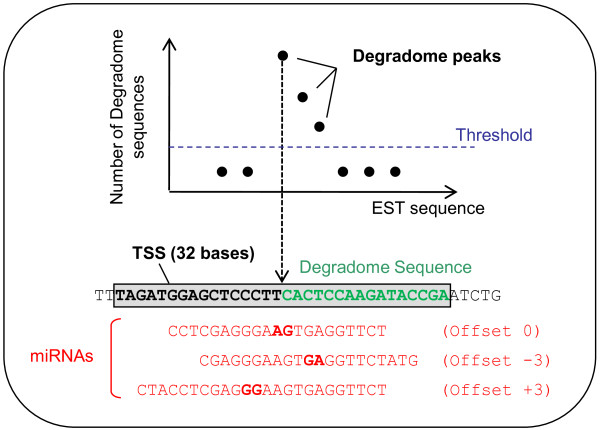
**Identification of miRNA regulated genes.** The degradome sequences were mapped to EST sequences. A threshold was determined for each EST based on the standard deviation from the average number of matching degradome sequences. Signatures with read numbers above the threshold were considered as degradome peaks and were used to determine Target Sequence Signatures (TSS). Each TSS was obtained from the EST by extracting the 32 nt sequence centred around the 5’end of a peak signature (cleavage site). The TSSs were used to search for miRNAs potentially responsible for the cleavage. The aligned miRNAs were regrouped depending on the offset (indicated in brackets) between their 10^th^ and 11^th^ nucleotide (bold characters) and the cleavage site. The targets were assigned to one of the three categories (Cat-I, II or III) depending on the number and offset distribution of the matching miRNAs.

The degradome sequences were mapped to the HarvEST dataset. The total reads mapping to an EST were used to establish a threshold which was calculated using the average number of reads of all degradome signatures matching the EST plus two standard deviations. Sequences that were more abundant than the threshold were considered to be degradome peaks (Figure 5). The degradome peak was then used to define a Target Signature Sequence (TSS, Figures 1 and 5) which extended 16 nt in each direction from the 5’ end of the degradome sequence that identified the peak. The TSSs were then compared to the known miRNAs, the new miRNAs and to the 19–23 nt smRNAs filtered against repeat elements (Figure 1; see Methods for detail). The three degradome libraries were analysed separately. This identified 1126 ESTs with at least one TSS that was predicted to be a binding site for a known, new or one of the 19–23 nt siRNAs. We refer to the 19–23 nt siRNAs that match a TSS as potential miRNAs (pot-miRNAs) from here on. As there is no precursor information for the pot-miRNAs they could include tasiRNAs and other siRNAs as well as genuine miRNAs. The majority of the ESTs we identified had multiple degradome peaks. As the degradome libraries were made by reverse transcription from the polyA + tail, a miRNA target would be expected to have a peak corresponding to the miRNA cleavage site, together with degradation products from downstream of the cleavage site. This prediction was observed for 17 out of 18 conserved targets of known miRNAs (data not shown). Based on this observation, we selected 96 ESTs for which the first TSS was predicted to be targeted by at least one miRNA (known, new or potential) that perfectly aligned with the predicted cleavage site (offset =0) (Additional file 4). These 96 ESTs included 21 with only one TSS and were targeted by a total of 1013 miRNAs (Additional file 4). Most of the TSSs were predicted to be targeted by multiple miRNAs; some were aligned to the predicted cleavage site, while others aligned at positions without a corresponding degradome product (offset of +/− 1 to 3). This last group of miRNAs, which do not appear to cleave the mRNA may be present in different tissues to the target mRNA. Another explanation could be that the precise position of the miRNA binding site on the target mRNA is critical for efficient cleavage by RISC due to structural constraints. As the presence of multiple miRNAs raises some doubt about the validity of the mRNA target site, they were assigned to three groups (Figure 5). Category I included the ESTs only targeted by miRNAs with a perfect offset. Category II contains ESTs targeted by a majority of miRNAs with a perfect offset. Category-III contains the ESTs where the aligning miRNAs with a perfect offset were in the minority.

Among the 96 potential miRNA targets, we found 17 targets of known miRNAs and three targets of new miRNAs (hvu-miR6005, hvu-miR6001, hvu-miR5071b) (Table 4, Additional file 4). The cleavage of three targets of known miRNAs was verified by RLM-5’RACE (Additional file 5). The pot-miRNAs identified in this analysis included many homologs of known miRNA families that varied in sequence and length to previously identified sequences (e.g. 71 miR156 homologs and 24 miR168 homologs). In the absence of complete genomic sequence data it is not possible to determine whether these represent genuine additional family members or errors from library construction and sequencing. The presence of large numbers of these alternate length sequences for some miRNA families suggests that there may be differential processing of the pri-miRNAs or later processing of the terminal nucleotides.

**Table 4:** Distribution of the potential miRNA targets by categories

**CATEGORIES**	**I**	**II**	**III**
Targets of pot-miRNAs	4	20	52
Targets of new miRNAs	1	2	
Targets of known miRNAs	11	2	4
Subtotal	16	24	56
Total	96		

The targets correspond to barley ESTs potentially regulated by miRNA mediated cleavage. The categories refer to a level of confidence based on the distribution of the smRNAs predicted to bind the target site (see Results and Discussion). The known miRNAs are those identified in previous studies, the new miRNAs are smRNAs for which we identified a pri-miRNA and the pot-miRNAs refer to the remaining set of 19–23 nt smRNAs matching to a TSS (Figure 1). Note that targets of known and new miRNAs may also be targeted by pot- miRNAs.

The degradome analysis revealed that most miRNAs target only one EST with a smaller group targeting 2 to 4 different ESTs, which are usually members of the same gene family, (e.g. ARFs, CBFs and SPLs; Additional file 4). This is in contrast to an average of 10 ESTs bioinformatically predicted as targets for each miRNA (data not shown). One obvious reason for not detecting target mRNA cleavage is that expression of the miRNA and mRNA may not overlap. Published microarray data [45,52] shows that a majority of the genes predicted to be regulated by a slicing mechanism are expressed during seed development (data not shown) however this does not preclude the non-overlapping expression of miRNA and target in the same cell types. In addition we found four sliced targets in our dataset that were predicted to be regulated through a translational repression mechanism only (Additional file 4). Our observations suggest that there are inadequacies in the algorithms currently used to predict miRNA targets, hence experimental verification is required to confirm these predictions *in planta*.

### miRNA regulation during seed development

**Figure 6:**
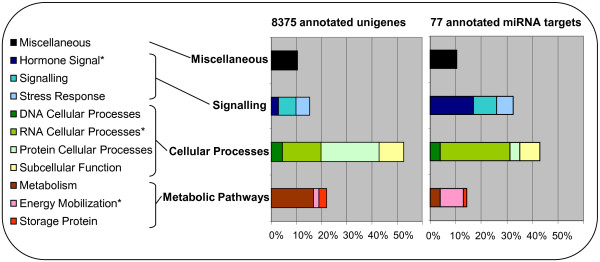
**Ontology of 77 miRNA targets*versus*8375 genes expressed in the seed.** Functional distribution of 8375 unigenes expressed in the seed and previously annotated [45] and 77 potential miRNA targets. The histograms present the percentage of genes involved in 11 biological processes that were grouped into 4 classes. *Categories significantly enriched (P < =0.01) in the miRNA target gene set based on a hypergeometric one-tailed test with correction for multiple testing (Benjamini-Hochberg).

The early development of the grain is controlled by a complex interaction of signalling and gene regulation networks to allow the proper expansion and specialisation of the different tissues that will constitute the mature grain. Based on our combined analysis of smRNA and degradome data we identified 96 genes likely to be miRNA regulated (by cleavage) during the first 15 DPA of seed development. Using annotated sequences from barley, wheat, rice and *Arabidopsis*, we found significant homology to an annotated gene for 77 of the miRNA target genes. Based on sequence homology these genes are predicted to encode a wide range of protein functions, including transcription factors, kinases, oxidoreductases, hydrolases, transferases, receptors and transporters (Additional file 4). We performed an ontology analysis of these targets and compared it to a set of over 8000 ESTs previously detected in the seed and annotated by Sreenivasulu *et al.* (Figure 6) [45]. Enrichment of GO terms was declared statistically significant if they met the criteria of P < = 0.01 using a hypergeometric one-tailed test with correction for multiple testing (Benjamini-Hochberg). This analysis shows that in the barley grain, miRNAs target a significantly higher percentage of genes annotated in the hormone signalling pathways, RNA cellular processes (which includes transcription factors) and energy mobilization categories.

Using our data, the variation of mature miRNA abundance was compared to that of the cognate degradation products across the three stages of grain development. The detection of mRNA cleavage products indicates that the expression domains of the miRNA and target gene are at least partially overlapping. The more the miRNA and its target are expressed, the more degradation products should be generated. The following paragraphs highlight what we think are the more interesting data based on the function of the targets. Since it is impossible to distinguish which one of the miRNAs (if not all) are present and functional in the same tissue as the target, all miRNAs with zero offset to the cleavage site were considered. The number of distinct miRNAs and the sum of their reads for each library is summarised in Table 5. As noted above, related miRNAs tend to have similar expression profiles and thus the sum of their reads is a good indication of their individual expression patterns.

**Table 5:** Potential miRNA target genes regulated during barley seed development

**EST**					**smRNAs with perfect offset**					**Degradome**		
**ID**	**Closest homolog**	**Sp.**	**E-score**	**Cat.**	**Family**	**nb**	**RPM-A**	**RPM-B**	**RPM-C**	**RPM-A**	**RPM-B**	**RPM-C**
	*Transcription factors*											
U21_18652	APETALA2-like protein	tae	0.E + 00	I	miR172	3	14.78	58.87	58.84	1.64	0.63	0.36
U21_19162	CBF (NF-YA2 homolog)	osa	8.E-80	I	miR169	2	4.10	1.49	0.78	1.02	0.30	1.60
U21_13817	CBF (NF-YA5 homolog)	osa	4.E-29	I	miR169	4	7.66	5.54	5.68	3.84	1.16	0.71
U21_8533	CBF (NF-YA9 homolog)	tae	5.E-40	II	miR169	2	4.10	1.49	0.78	7.38	2.41	3.48
U21_9757	NAC	osa	1.E-126	I	miR164	20	324.78	560.70	314.03	0.72	0.83	0.82
U21_3667	GAMyb protein	hvu	0.E + 00	I	miR159	5	6.44	4.46	3.35	6.07	6.84	2.28
U21_18637	SPL16	osa	6.E-83	I	miR156	77	4418.37	8965.97	7953.70	1.48	0.03	0.21
U21_19856	SPL18	osa	2.E-52	I	miR156	77	4418.37	8965.97	7953.70	2.00	1.06	0.14
U21_13717	DOF1	tae	1.E-154	II	**pot-miRNA**	3	25.59	29.03	35.61	0.39	0.00	0.00
	*Chloroplast functions*											
U21_4432	RNP-A, chloroplastic	osa	1.E-112	II	**pot-miRNA**	1	1.37	1.08	0.77	1.18	0.17	0.00
U21_495	Carbonic anhydrase, chloro.	osa	1.E-115	III	**pot-miRNA**	1	1.64	1.08	1.16	0.26	0.40	1.56
U21_40749	Chlorophyll a-b binding prot.	tae	3.E-40	II	**pot-miRNA**	3	3.28	1.22	1.55	0.00	0.40	0.00
U21_1307	Ferredoxin, chloroplastic	tae	1.E-54	III	**pot-miRNA**	2	2.33	1.08	1.03	91.01	112.87	75.62
U21_6135	PGlcT	ath	9.E-40	I	**pot-miRNA**	1	0.41	0.68	0.10	0.53	8.32	8.46
	*Phytohormone signalling*											
U21_11104	VP1/ABI3	osa	4E-90	III	miR516	2	2.05	6.08	6.19	0.00	0.00	0.78
U21_48909	ABI8	tae	1E-71	III	**pot-miRNA**	5	15.06	15.53	22.71	0.00	0.20	0.00
U21_9888	GA 3-oxidase 1	hvu	0.E + 00	I	**pot-miRNA**	7	145.90	168.22	129.92	0.00	0.23	0.14
U21_9040	GID1	hvu	1E-133	II	**pot-miRNA**	8	3.83	37.94	114.57	0.49	0.07	0.00
U21_7409	TIR1	osa	9.E-231	III	miR393	1	0.14	0.81	0.90	3.64	4.26	2.52
U21_12845	ARF1 (AtARF6-like)	osa	1.E-119	I	miR167	37	1424.78	2234.51	2583.93	3.91	1.82	2.63
U21_8147	ARF2 (AtARF6-like)	osa	8.E-176	I	miR167	37	1424.78	2234.51	2583.93	3.91	1.82	2.63
U21_19718	ARF3 (AtARF6-like)	osa	1.E-116	I	miR167	37	1424.78	2234.51	2583.93	3.91	1.82	2.63
U21_1664	ARF4 (AtARF2-like)	osa	5.E-302	II	tasiR-ARFs	8	299.60	129.61	36.00	0.82	0.13	0.00
U21_19004	ARF5 (AtARF3-like)	osa	1E-56	II	tasiR-ARFs	8	299.60	129.61	36.00	0.26	0.20	0.00
U21_24760	ARF6 (AtARF3-like)	tae	1E-119	II	tasiR-ARFs	8	299.60	129.61	36.00	1.12	0.50	0.00
U21_26602	ARF7 (AtARF3-like)	tae	1E-124	III	tasiR-ARFs	3	49.68	14.18	4.39	3.25	0.43	0.64
U21_22467	ACC oxidase	osa	1E-24	II	**pot-miRNA**	1	2.05	2.16	0.90	0.00	0.17	0.00
	*Defence response*											
U21_18842	OsMLA10-like	osa	1E-47	I	miR5071	23	651.63	537.46	257.39	0.98	0.40	0.00
U21_23305	OsMLA1-like	osa	1E-72	III	**pot-miRNA**	4	35.31	28.76	22.19	3.97	0.86	1.17

List of 29 of the 96 potential miRNA targets identified in this study. The left part of the table shows ESTs predicted to be regulated through miRNA-mediated cleavage, their category of confidence (cat.), and their associated function according to homology with the closest annotated gene from one of the following species (sp.): *Hordeum vulgare* (hvu), *Triticum aestivum* (tae), *Oriza sativa* (osa) or *Arabidopsis thaliana* (ath). Genes are arranged by functional group indicated by the headings in the closest homolog column. The middle part of the table shows the number (nb) of smRNAs matching the target with a perfect offset, the sum of their reads (in RPM) in each library and to which family they belong (pot-miRNA indicates when no match was found to a known or new miRNA identified in this study). The right part shows the number of degradome sequences (in RPM) resulting from the cleavage. The grey boxes indicate when the number of reads was significantly higher than the threshold.

### Cell differentiation

Perhaps the best known function of the miRNA pathway is to control the cell fate through the regulation of transcription factor coding genes. We validated 11 conserved targets of six known miRNA families which code for transcription factors known to control key steps in plant development: miR156-*SPL* (2 genes), miR159-*Myb*, miR164-*NAC*, miR167-*ARF* (potentially 3 genes sharing the same degradome peak), miR169-*CBF* (3 genes) and miR172-*AP2like* (Table 5). We also found evidence for miRNA regulation of the DOF (DNA binding with one finger) plant specific transcription factor family. Its expression seems to be restricted to the early development of the grain since degradation products were observed only during stage A (Table 5). DOFs are plant specific transcription factors known to play a critical role in growth and development [53]. In maize and finger millet, DOF proteins are thought to be involved in carbon metabolism and the accumulation of storage proteins [54,55]. In rice, RPBF (rice prolamin box binding factor) which contains a DOF domain, was shown to be involved in the regulation of endosperm expressed genes [56].

### Energy mobilization

The early development of the seed is associated with an elevated metabolic activity limited by energetic resources. Photosynthesis related genes are mainly expressed during the first 5 DPA within the pericarp tissue [43]. Four of the potential miRNA targets (encoding a ferredoxin, a chlorophyll a/b binding protein, a carbonic anhydrase and a ribonuclear protein) are likely to be involved in chloroplast function. An EST coding for a PGlcT (Plastidic GlucoseTranslocator) homolog is also cleaved by a pot-miRNA during the early development of the grain. PGlcT is involved in the export of stored starch into the cytoplasm at night [57]. The level of PGlcT degradation products in our dataset increases during grain development (Table 5) which correlates with a previous observation in rice that expression of a PGlcT homolog gene increases in the endosperm during the first 15 DPA [58].

### Signalling pathways

The control of seed development involves a cross-talk between three key phytohormones: ABA, GA and auxin, which are tightly linked to the master regulators LEC1/AFL (LEC1: Leafy Cotyledon1 and AFL - referring to B3 domain factors: ABI3, FUS3 and LEC2) that govern many seed-specific traits, such as embryogenesis, grain filling, desiccation tolerance, and dormancy induction [59-62].

Auxin concentration together with other local factors, contributes to cell differentiation and specification of cell fate [63,64] and is known to be involved in embryo patterning [65]. In *Arabidopsis*, the auxin signal is tightly linked to the miRNA pathway, with four conserved miRNA families (miR160, miR167, miR390 and miR393) regulating the auxin receptor *TIR1* (*Transport Inhibitor Response1*) and different subgroups of *ARF* (*Auxin Response Factor*) genes [9,66-71]. We identified a *TIR1 homolog* and 7 *ARF* genes potentially regulated by miRNAs and tasiRNAs during seed development (Table 5). Our data shows that in the barley grain the regulation of *TIR1* and potentially 3 *ARF* genes (the same degradome peak matches three distinct ESTs) by the miR393 and miR167 families is conserved. We noticed that hvu-miR167a and d, which are the highest expressed members in this family, show a reciprocal accumulation pattern which could suggest they are expressed in different tissues where they differentially regulate the same target genes (Additional file 4). We also identified smRNAs homologous to the tasiR-ARFs which regulate four *ARFs* (*ARF 4/5/6/7*). The accumulation of these smRNAs correlates with hvu-miR390 which gradually decreases in abundance from stage A to C (Table 5, Additional file 2), suggesting that, as in *Arabidopsis*, the production of the tasiR-ARFs requires miR390-mediated cleavage.

The antagonistic role of GA and ABA in the control of the switch between dormancy and germination is a well known mechanism; however the function of these hormones during the early stages of seed development remains unclear. Our data suggest that there is miRNA regulation of ABA and GA signalling during the early stages of grain development, with degradome analysis identifying two *ABA-Insensitive* homolog genes (*ABI3* and *ABI8*), a *GA3oxidase1* and a homolog of the GA receptor *GID1* (*Gibberellin Insensitive Dwarf1*) as targets of miRNAs or pot-miRNAs (Table 5). *ABI3* is cleaved with a perfect offset by 2 members of the grass specific miRNA family hvu-miR516. Degradation products of *ABI3* only accumulate during stage C whereas the corresponding miRNAs are expressed earlier. In contrast, the cleavage of *GID1* mostly occurs during early stages while the cognate pot-miRNAs accumulate in later stages. This suggests that in both cases the miRNAs could act to prevent leakage in target gene expression, ensuring that *GID1* function is restricted to early stages and *ABI3* to later stages. These data support the current belief that GA is required during early embryogenesis but its function is repressed in later phases when a higher ABA/GA balance is needed for the proper maturation of the grain. This also correlates with the observation that the late presence of GA may inhibit embryogenic cell differentiation [72].

Our data also suggest that there is miRNA regulation of ethylene responses with an *ACC* oxidase homolog cleaved by a pot-miRNA during the early maturation phase. Along with ABA, ethylene is thought to play a major role in the development of the endosperm by affecting grain filling and the timing of programmed cell death (PCD) [44,46].

### Defence response

Plants recognize many pathogens through the action of a diverse family of R genes, whose protein products are necessary for the direct or indirect recognition of pathogen avirulence (avr) proteins in order to initiate the defence response. In addition to their role in defence responses, R genes may be involved in the regulation of developmental processes in *Arabidopsis* and rice [73,74].

In barley, the R gene *MLA10* acts as a receptor of fungal infection by recognising avirulence proteins and confers resistance against the powdery mildew fungus [75,76]. In wheat the expression of several miRNAs is responsive to powdery mildew infection, suggesting that the miRNA pathway could be involved in triggering the defence response [77,78]. Our degradome analysis indicates that two *HvMLA* genes homologous to the rice *MLA1* and *MLA10* genes are cleaved by miRNAs or pot-miRNAs (Table 5).

### Further investigation of *OsMLA10-like* and *GA3oxidase1* regulation

The impact of regulation by a miRNA depends on the relative spatio-temporal accumulation of the miRNA and the target mRNA. In this study, we focused on investigating miRNA regulation at three consecutive stages of grain development. Since the tissues that will constitute the mature grain are not formed during the early stages, we used the whole caryopsis to be able to compare the abundance of the mature miRNAs and the degradation of their targets between the samples. Consequently, further investigation of the function of a miRNA cleavage identified in our analysis requires further assessment of the tissue specificity of both miRNA and target mRNA expression.

**Figure 7:**
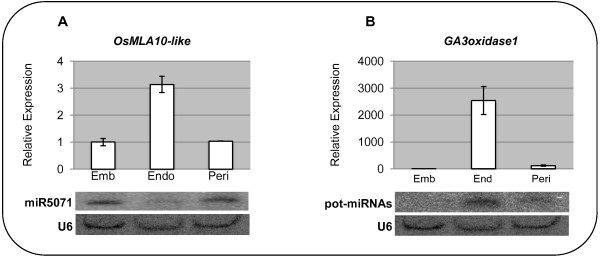
**Tissue specificity of*OsMLA10-like, GA3oxidase1*and their respective miRNAs.** Upper panels show the quantification by RT-qPCR of the mRNA levels of: **A**. *OsMLA10-like* and **B**. *GA3oxidase1*, in different caryopsis tissues (embryo, endosperm, pericarp). The values presented are an average of 3 biological replicates. Lower panels show the detection by northern blot of the corresponding miRNAs. Tissues were harvested during stage C (11–15 DPA). U6 was used as loading control for small RNA blots.

To illustrate this, the regulation of two category-I targets, *OsMLA10-like* and *GA3oxidase1*, and their associated miRNAs was investigated. The abundance of both miRNAs and targets were quantified in embryo, endosperm and pericarp tissues dissected from the caryopsis at stage C (Figure 7). For *OsMLA10-like* in these tissues, the corresponding miRNAs (which belong to the miR5071 family) are mostly detected in the embryo and pericarp whereas *OsMLA10-like* expression is higher in the endosperm. The degradation products detected during earlier stages suggest that *OsMLA10-like* transcription was initially higher and that the function of the miRNA was to inhibit its expression in the embryo and pericarp. In contrast, the pot-miRNAs targeting the *GA3oxidase1* gene predominantly accumulate in the endosperm where the target is also actively transcribed. According to the degradome data, the *GA3ox1* probably starts to be expressed during stage B when the first cleavage products can be detected. The role of the miRNA(s) may be to modulate level of *GA3ox1* transcripts and consequently prevent excess GA accumulation in the endosperm. These two examples highlight the complexity of multilayer gene regulations and the requirement for complementary studies in order to analyse how, where and when a gene is regulated.

## Conclusion

The data we have generated provides a comprehensive source of information about the timing of miRNA regulation during grain development. Regulation by miRNAs peaks during the transition phase (5–10 DPA) which correlates with the timing of a major change in transcript profiles. The 96 potential miRNA target genes we identified are predicted to be involved in various functions including photosynthesis, carbohydrate translocation, phytohormone signalling, cell differentiation and defence response. Our data suggest an upstream function of the miRNAs in coordinating tissue specification and energy mobilization to ensure proper growth and development of the grain.

As increasing amounts of genome sequence data become available our data can be re-examined to identify more miRNA precursors and refine the predictions of which genes are under miRNA regulation. The analysis of the biological roles of miRNAs in cereals currently depends on transgenic approaches; however the identification of miRNA resistant target mRNAs that give rise to altered phenotypes in rice and barley [34,35,38] suggests that the use of high-throughput methods to identify sequence changes leading to miRNA-resistant targets will allow assessment of the roles of other miRNAs.

## Methods

### Biological material

Barley (*Hordeum vulgare*) plants were grown in naturally lit phytotron glasshouses with air temperature set at 17°C/9°C day/night cycle. The plants were grown from October to December when the time of anthesis was determined for each head based on the dissection of the middle spikelet. Immature grains were harvested from the middle six rows of the head at 1 to 15 Days Post Anthesis (DPA). Total RNA was extracted from 100 mg of seeds from each DPA (which correspond to ~50 seeds at 1 DPA and 2 seed at 15 DPA) using the following method. Whole caryopsis was ground in a mortar using liquid nitrogen. 1.2 mL of NTES buffer [NaCL 100 mM, Tris-pH8.0, 10 mM, EDTA 1 mM, 1%(w/v) SDS] and 1.6 mL of phenol:chloroform:isoamyl alcohol [25:24:1] were added in the mortar and grinding continued until tissue was thawed. The extract was centrifuged 5 min at 12,000 rpm. The supernatant was precipitated by adding 1/10 vol of 3 M NaOAc and 2.5 vol of 100% ethanol and incubating at −20°C overnight. The extract was centrifuged 20 min at 4°C and 12,000 rpm. The pellet was washed in 75% ethanol, centrifuged 5 min at maximum speed, dried for 1 min at room temperature and resuspended into 50 μl of RNAse-free water. Samples were DNase treated using RQ1 DNase from Promega for 20 min at 37°C followed by a phenol:chloroform extraction and a second ethanol precipitation. Total RNA extracts were resuspended into 50 μL of RNAse-free water and the quality was determined using a Nanodrop spectrophotometer and agarose gel electrophoresis. An equal quantity of each RNA extract was pooled to constitute the following three samples: A (RNA extracts 1, 2, 3, 4, 5 DPA), B (RNA extracts 6, 7, 8, 9, 10 DPA), and C (RNA extracts 11, 12, 13, 14, 15 DPA).

### Construction and analysis of smRNA libraries

To determine the smRNA populations present in samples A, B and C, 60 μg of total RNAs were used to prepare libraries for Illumina sequencing (http://www.geneworks.com.au). A custom script was used to trim reads of 3’ adapter sequences and then to pool identical reads to create a non-redundant sequences (=signatures) list. Sequences that were over 50% homopolymer or dinucleotide repeat were filtered out. Read counts for each sequence were normalised to reads per million of total sequenced (RPM). The diversity of the signatures present in the three libraries had a high number of singletons, over 80% (Table 1). To facilitate the analysis and build a level of confidence from the sequences cloned in the libraries, signatures were only considered that were 18 to 25 nucleotides in length and with a minimum expression of 1 RPM, representing 137,614 smRNAs. For identification of previously known miRNAs, signatures were checked for an exact match (in sequence and length) to a known miRNA present in miRBase [10] or recently in barley leaves [16]. To identify new miRNAs, the signatures of 19 to 23 nucleotides in length were kept and aligned to the HarvEST unigene set (release-21, http://www.harvest-web.org/) using SOAP [79]. Signatures with no more than 20 matching unigenes and with no match to a smear of overlapping smRNA sequences were kept. For near identical sequences aligning to the same location (for example, differing in length by a base or being offset by a base) only the sequence with the highest read count was retained. The miRNA precursors were searched by extracting the unigene sequence surrounding the aligned smRNAs and testing their potential to form a hairpin secondary structure using Vienna RNALFold (http://www.tbi.univie.ac.at/~ivo/RNA/). The smRNA signature was required to have no more than 4 mismatches against the complementary sequence in the hairpin structure and no more than 2 bulges. Considering that MIR genes may originate from the evolution of an inverted repeat element that initially can produce endogenous 24 nt-siRNAs [80,81], we kept the precursors sharing less than 70% homology within the pre-miRNA region to repeat elements in the Plant Repeat Databases [82]. smRNA which could be found in a stem of a potential miRNA precursor-like hairpin structure in the folded sequences were marked as new miRNAs. For the downstream analysis of miRNA target genes, the remaining 19–23 nt signatures with no more than 90% homology to a repeat element were kept (Plant Repeat Databases [82], Figure 1).

### Construction and analysis of degradome libraries

The mRNA degradome libraries were made as described by German *et al.*[83] using total RNA extract from the 3 samples A, B and C. In brief, for each sample, 200 μg of total RNA was used to purify messenger RNA using an mRNA purification kit (Stratagene #400806). A 5’-adaptor (5′-GUUCAGAGUUCUACAGUCCGAC-3′) was linked to the mRNA 5’CAP-less fragments and purified again using the mRNA purification kit. After reverse transcription using the RT-primer (5'-CGAGCACAGAATTAATACGACTTTTTTTTTTTTTTTTTTV-3'), the cDNAs were amplified through 7 PCR cycles using the primers P1 (5'-GTTCAGAGTTCTACAGTCCGAC-3') and P2 (5'-CGAGCACAGAATTAATACGACT-3'). Amplicons were digested by *Mme*I (New England Biolabs, #R0637S) and dephosphorylated by Shrimp Alkaline Phosphatase treatment (Roche #11758250001). Samples were run on a 12% polyacrylamide gel and the *Mme*I cleaved fragments corresponding to the 42 bp gel band were purified. Purified Products were ligated to a double stranded DNA adaptor (5'-P-TCGTATGCCGTCTTCTGCTTG-3' + 3'-NNAGCATACGGCAGAAGACGAAC-5') and purified on a second 12% polyacrylamide gel by extracting the 63 bp gel band. The DNA fragments were amplified by 21 PCR cycles using the primers P3 (5'-AATGATACGGCGACCACCGACAGGTT-CAGAGTTCTACAGTCCGA-3') and P4 (5'-CAAGCAGAAGACGGCATACGA-3'), and purified again on 12 % polyacrylamide gel by excising the 86 bp band. The purified amplicons, which constitute the degradome libraries, were sequenced using the Illumina platform.

As for the small RNA analysis, reads were trimmed, reduced to a non-redundant set, filtered for repetitive sequence and their read counts were normalised in read per million (RPM). For subsequent analysis, sequences of 21 nt in length were trimmed back to 20 nt, then only sequences of 20 nt in length and having at least 1 RPM were retained. Kanga (http://code.google.com/p/biokanga/) was used to align the 20 nt signatures to the HarvEST unigene sequences (release-21, http://www.harvest-web.org/). No mismatches were allowed in the alignment. For each matching EST the number of aligned degradome sequences at each position along the EST was investigated to identify signature peaks (Figure 5). Positions for which the number of aligned sequences exceeded the mean plus two standard deviations for a sample along an EST were retained. From each of these retained signature peaks, a 32 nt sequence was extracted from the EST, centred around the 5' end of the aligned signature, to constitute the Target Signature Sequence (TSS). To identify the smRNAs that could potentially bind to a TSS we used psRNAtarget (http://plantgrn.noble.org/psRNATarget). We ran the known miRNAs, new miRNAs and 19–23 nt smRNAs against the TSS with a maximum expectation of 5 and an hspsize (which is the length of the region used to score the complementarity between the miRNA and its target) equal to the length of the smRNA (to ensure that the entire sequence of the smRNA is considered by the scoring algorithm). We indicated the offset between the predicted cleavage site of the smRNA (position 10-11nt of the smRNA) and the detected cleavage site (center of the TSS, Figure 5), using the formula: offset = detected cleavage position on the EST - predicted cleavage position on the EST. For each TSS, we kept all smRNAs in a −3/+3 offset window. Since the smRNAs with a common 3’ end binding position on the EST share the same predicted cleavage site we considered them as one group and categorized the targets according to the offset distribution of these smRNA groups (Table 4). We categorized the targets as follows: category-I; the ESTs targeted by a unique smRNA group with a perfect offset (considered as unique when the smRNA group represented more than 97% of the total number of smRNAs predicted to bind to the TSS), category-II; the ESTs targeted by a majority of smRNAs with a perfect offset and category-III; the remaining ESTs targeted by a minority of smRNAs with a perfect offset.

### Quantitative RT-PCR

RT-PCR reactions were performed as previously described [40]. In brief, first-strand cDNA was synthesized using oligo(dT) primers and Super-Script III reverse transcriptase (Invitrogen). PCR reactions were performed on an AB 7900 HT Fast Real-Time PCR System (Applied Biosystems). 1.0 μL of 1:10 diluted template cDNA was used in a 10 μL reaction. The amplification program was: 1 cycle of 15" at 95°C, 35 cycles 15" at 95°C, 30" at 60°C, 30" at 72°C, and then followed by a thermal denaturing step. All primers pairs of the tested genes showed a similar amplification efficiency to the one used for the *ACTIN* gene which was used as reference. Relative transcript levels of biosynthesis were calculated with the ΔΔCt method (Applied Biosystems). Forward and Reverse primers: HvDCL1a F (AGAAGCCTTGACTGCTGCAT) and R (ATCAATTTCGCCCTCCTCTT); HvDCL1b F (GCCCCAAAAGTGCTATCTGA) and R (GCCCCGACATCTCCTTTAGT); HvDCL1c F (CGGCAGAAACAATTGATGAG) and R (CAAAGCTTCCTGTTGCACTG); GA3ox1 F (GCACTACCGCCACTTCTCTG) and R (CTCTCGGTGAGGTTGTGCTC); OsMLA10-like F (ATAAGATACGTCGTCTGTCCATG) and R (TCCAACACCCGCAGAGCATG).

### Northern blot analysis

Total RNA (40 μg) was separated on a denaturing 15% polyacryamide gel containing 7 M urea at 120 V for 2 hr. RNA was electrophoretically transferred to Zeta-probe GT membranes (BioRad) at 40 V for 90 min and fixed by UV crosslinking. Membranes were incubated in hybridization buffer [Na_2_PO_4_-pH7.2 125 mM, NaCl 250 mM, 7%(w/v) SDS, 50%(v/v) formamide] for 4 h at 42°C and then incubated in the presence of ^32^P-end-labeled oligonucleotide probes at 42°C overnight. Membranes were washed in [2X SSC, 0.2% SDS] at 42°C and radioactivity was detected using a Phosphorimager. Oligonucleotide probes: miR164-AS (5’-TGCACGTGCCCTGCTTCTCCA-3’), miR168-AS (5’-GTCCCGATCTGCACCAAGCGA-3’), miR390-AS (5’-GGCGCTATCCCTCCTGAGCTT-3’), miR-MLA10-AS (5’-GGTCCATGATATGATGC[45]TTGA-3’), miR-GA3ox1-AS (5’-TCCACTGAGCTACAGGCGC-3’).

### RLM-5’ RACE

RNA ligase-mediated 5′ rapid amplification of cDNA ends (RLM 5′-RACE) was performed using the GeneRacer kit (Invitrogen). The manufacturer’s protocol for 5’end analysis was followed with the exception of the 5’ de-capping step. In brief, total RNA was isolated from whole caryopsis tissues at 6–10 DPA and ligated to a 5’end RNA adaptor before being reverse transcribed using an oligo(dT) primer. The PCR reactions were performed using the following gene specific reverse primers: U21_3667-R (GGGGACTGCATGTACGGATC), U21_18637-R (GAGACGGTGCCGGTGGAAGCCT) and U21_9757-R (AGACATGCTCGGCACCACCTCACCA).

The small RNA and degradome sequence datasets have been deposited in the NCBI GEO database, accession GSE38755.

## Abbreviations

miRNA, microRNA; GA, Gibberellic acid; ABA, Abscisic acid; PARE, Parallel analysis of RNA ends; RACE, Rapid amplification of RNA ends; DCL, Dicer-like.

## Competing interests

The authors declare no competing financial interests.

## Authors’ contributions

JC designed the study, carried out experiments, analysed data and drafted the manuscript. AS analysed data. JT advised on data analysis. ZL helped design the study and draft the manuscript. CH designed the study and helped draft the manuscript. All authors read and approved the final manuscript.

## Supplementary Material

Additional file 1 **Composition of the smallRNA and mRNA degradome libraries (Excel file).** Total number of sequences and signatures (unique sequences) for each length of cloned sequence found in smRNA and mRNA degradome libraries made from whole caryopsis tissue at the three developmental stages A (1–5 DPA), B (6–10 DPA) and C (11–15 DPA). Click here for file

Additional file 2 **List of the known miRNAs identified in the barley grain (Excel file).** List of the 84 previously identified miRNAs that were found in the barley grain. RPM-A/B/C shows their abundance in Read Per Million in the smRNA libraries made from whole caryopsis tissue at the three developmental stages A, B, C. The column "leaf" indicates if the miRNAs was found in barley leaf by Schreiber et al., 2011 [16]. The specificity of each miRNA was determined according to its conservation across the plant kingdom. miRNA homologs are shown in the last column using the nomenclature from miRBase, except for Hv-Sc-miRNA refering to miRNAs found by Schreiber et al., 2011 [16]. The number in brackets indicates the number of mismatches with the sequence found in our smRNA libraries. (XLS 48 kb) Click here for file

Additional file 3 **miRNA precursors sequences and MFOLD structures (Excel file).** List of the 15 pri-miRNAs identified in the harvEST dataset. The yellow boxes indicate the newly identified pri-miRNAs and mature miRNAs. RPM-A/B/C show the abundance of each miRNA (RPM) in the smRNA libraries made from whole caryopsis tissue at the three developmental stages A, B and C. miRNA* indicated a corresponding miRNA* found in the libraries. The columns "Start" and "End" show the position of the miRNA in the EST sequence. The predicted secondary structure of the pri-miRNA sequences were assessed using MFOLD (http://mfold.rna.albany.edu). The nucleotides corresponding to the miRNAs are shown in red and those corresponding to the miRNA* (if detected) are shown in blue. (XLS 53 kb) Click here for file

Additional file 4 **List of the potential miRNA target genes and their associated miRNAs (Excel file).** List of 96 potential miRNA targets regulated in the barley grain by 1013 miRNAs. Target information (left part of the table) includes the EST identity (ID); the category of confidence based on the smRNA distribution around the cleavage site; the proposed molecular and biological function according to homology with the closest annotated gene from one of the following species (sp.): Hordeum vulgare (hvu), Triticum aestivum (tae), Oryza sativa (osa) or Arabidopsis thaliana (ath). Degradome information (middle part of the table) shows the Target Sequence Signature (TSS) centered around the detected cleavage site; the number of corresponding mRNA degradation products (in Reads Per Million) found in the degradome libraries made from whole caryopsis tissue at the three developmental stages (A, B, C) with an "*" indicating when the degradome products were significantly higher than the threshold (threshold was calculated using the average number of reads of all degradome signatures matching to the EST plus two standard deviations (SD) for each sample); the total number of smRNAs matching the TSS. miRNA information (right part of the table) shows the length, sequence and number of reads of all smRNAs that can potentially bind to the TSS with a maximum score of 5 (note that the pot-miRNAs include all 19–23 nt smRNAs that can bind to the TSS, such as tasiRNAs); the number of target for each miRNA; the type of inhibition predicted by psRNAtarget. (XLS 692 kb) Click here for file

Additional file 5 **Results of RLM-5’RACE for three targets of known miRNAs.** The sequences correspond to the 36 bp TSS of each target; the base in green shows the 5’end position of the corresponding degradome signature. Numbers in red refer the ratio of 5’-RACE clones matching the site indicated by an arrow over the total number of clones sequenced. (PPT 134 kb) Click here for file
