# Supplementary material for: miRNA regulation in the early development of barley seed
Source: BMC Plant Biol. 2012 Jul 28;12:120. doi: 10.1186/1471-2229-12-120 (PMC3443071; doi:10.1186/1471-2229-12-120)
Supplement: Additional file 5 — Results of RLM-5’RACE for three targets of known miRNAs. The sequences correspond to the 36 bp TSS of each target; the base in green shows the 5’end position of the corresponding degradome signature. Numbers in red refer the ratio of 5’-RACE clones matching the site indicated by an arrow over the total number of clones sequenced. (PPT 134 kb) [file 1471-2229-12-120-S5.ppt]

## Slide 1
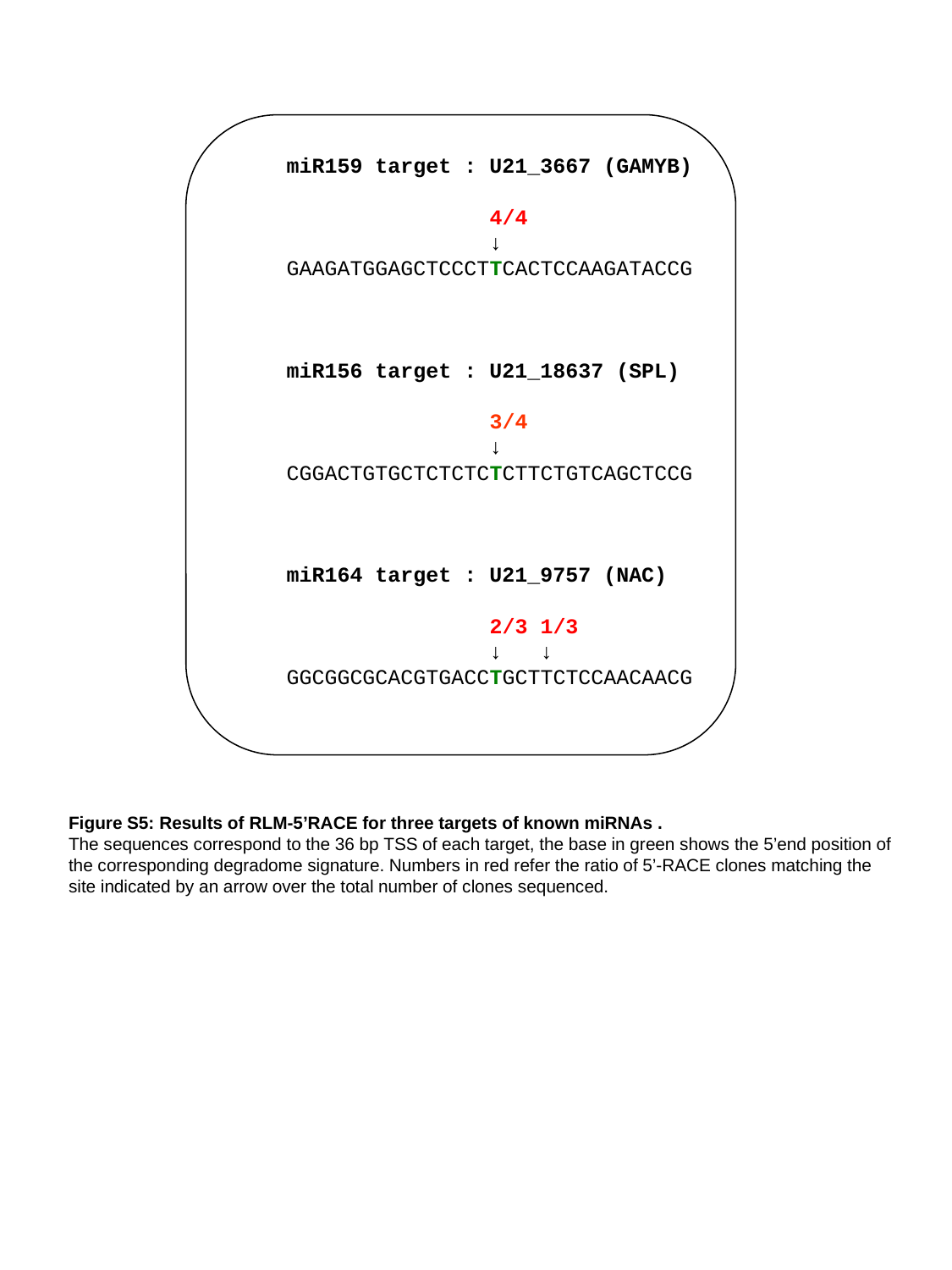

# miR159 target : U21_3667 (GAMYB)
 4/4
 ↓
GAAGATGGAGCTCCCTTCACTCCAAGATACCG
miR156 target : U21_18637 (SPL)
 3/4
 ↓
CGGACTGTGCTCTCTCTCTTCTGTCAGCTCCG
miR164 target : U21_9757 (NAC)
 2/3 1/3
 ↓ ↓
GGCGGCGCACGTGACCTGCTTCTCCAACAACG
Figure S5: Results of RLM-5’RACE for three targets of known miRNAs .
The sequences correspond to the 36 bp TSS of each target, the base in green shows the 5’end position of the corresponding degradome signature. Numbers in red refer the ratio of 5’-RACE clones matching the site indicated by an arrow over the total number of clones sequenced.
